# Not All Competitions Come to Harm! Competitive Biofeedback to Increase Respiratory Sinus Arrhythmia in Managers

**DOI:** 10.3389/fnins.2020.00855

**Published:** 2020-08-31

**Authors:** Elisabetta Patron, Marianna Munafò, Simone Messerotti Benvenuti, Luciano Stegagno, Daniela Palomba

**Affiliations:** ^1^Department of General Psychology, University of Padua, Padua, Italy; ^2^Padova Neuroscience Center, University of Padua, Padua, Italy

**Keywords:** competition, biofeedback, managers, respiratory sinus arrhythmia, autonomic nervous system

## Abstract

Despite the positive impact on achievement, competition has been associated with elevated psychophysiological activation, potentially leading to a greater risk of cardiovascular diseases. Competitive biofeedback (BF) can be used to highlight the effects of competition on the same physiological responses that are going to be controlled through BF. However, it is still unknown whether competition could enhance the effects of respiratory sinus arrhythmia (RSA)-BF training in improving cardiac vagal control. The present study explored whether competitive RSA-BF could be more effective than non-competitive RSA-BF in increasing RSA in executive managers, who are at higher cardiovascular risk of being commonly exposed to highly competitive conditions. Thirty managers leading outstanding private or public companies were randomly assigned to either a Competition (*n* = 14) or a Control (*n* = 16) RSA-BF training lasting five weekly sessions. Managers in the Competition group underwent the RSA-BF in couples and each participant was requested to produce a *better* performance (i.e., higher RSA) than the paired challenger. After the training, results showed that managers in the Competition group succeeded in increasing cardiac vagal control, as supported by the specific increase in RSA (*p* < 0.001), the standard deviation of R-R wave intervals (SDNN; *p* < 0.001), and root mean square of the successive differences between adjacent heartbeats (rMSSD; *p* < 0.001). A significant increase in the percentage of successive normal sinus beat to beat intervals more than 50 ms (pNN50; *p* = 0.023; η^2^*_*p*_* = 0.17), low frequency (*p* = ≤ 0.001; η^2^*_*p*_* = 0.44), and high frequency power (*p* = 0.005; η^2^*_*p*_* = 0.25) emerged independently from the competitive condition. Intriguingly, managers who compete showed the same reduction in resting heart rate (HR; *p* = 0.003, η^2^*_*p*_* = 0.28), systolic blood pressure (SBP; *p* = 0.013, η^2^*_*p*_* = 0.20), respiration rate (p < 0.001; η^2^*_*p*_* = 0.46), and skin conductance level (SCL; *p* = 0.001, η^2^*_*p*_* = 0.32) as non-competitive participants. Also, the same reduction in social anxiety (*p* = 0.005; η^2^*_*p*_* = 0.25), state (*p* = 0.038, η^2^*_*p*_* = 0.14) and trait anxiety (*p* = 0.001, η^2^*_*p*_* = 0.31), and depressive symptoms (*p* = 0.023, η^2^*_*p*_* = 0.17) emerged in the two groups. The present results showed that managers competing for increasing RSA showed a greater improvement in their parasympathetic modulation than non-competing managers. Most importantly, competition did not lead to the classic pattern of increased psychophysiological activation under competitive RSA-BF. Therefore, competition could facilitate the use of self-regulation strategies, especially in highly competitive individuals, to promote adaptive responses to psychological stress.

## Introduction

Competition has been consistently referred to as a type of social motivation, and, it has been addressed in many fields, including sports, job-related productivity, and academic achievement. Indeed, under challenging conditions, competition can motivate individual behavior more than cooperation. Certainly, competition contributes strongly to achievement-oriented behavior (Lam et al., 2004) by enhancing both competitor’s intrinsic motivation (Tauer and Harackiewicz, 2004), creativity (Baer et al., 2010), and by fostering the mastery of a skill (Cooke et al., 2011). In modern work environments, especially among high-level managers and leaders, competition is embraced to reach a high work pace and top efficiency (Zhu and Zhou, 2014).

On the other side of the coin, competition represents a considerable source of social pressure, leading to aversive emotional states (Baumeister and Showers, 1986; Cerin et al., 2000; Martinent et al., 2012) and promoting psychophysiological activation. Under competitive conditions, increased psychophysiological activation, supported by large sympathetic nervous system responses, especially involving the cardiovascular system, have been commonly reported (Harrison et al., 2001; van Zanten et al., 2002). Faster heart rate (HR) and a shortening of the pre-ejection period (an index of increased myocardial contractility), both markers of beta-adrenergic activation and reduced parasympathetic cardiac modulation (as measured by the root mean square of the successive differences between adjacent heartbeats; rMSSD), have been reported during competitive conditions independently of individuals competitiveness trait (van Zanten et al., 2002). Nonetheless, individuals with high competitive traits were found to show higher blood pressure (BP) reactions and greater shortening of the pre-ejection period during a competitive condition compared to non-competitive individuals (Harrison et al., 2001). More recently, Cooke et al. (2011) reported that competition elicited cardiac beta-adrenergic activation (as measured by a shortening of the R-wave to pulse interval), alpha-adrenergic activation of the vasculature (as measured by decreased pulse amplitude), and decreased total heart rate variability (HRV) as measured by the standard deviation of R-R intervals (SDNN). Intriguingly, the authors reported that a decrease in SDNN mediated the improvement in endurance performance during competition (Cooke et al., 2011). Altogether, these results suggest that competitive conditions induce a psychophysiological activation that seems to be supported by a cardiac parasympathetic withdrawal co-occurring along with sympathetic activation (van Zanten et al., 2002; Cooke et al., 2011).

Such an important psychophysiological activation during competition, while being a powerful factor in achievement motivation, is also a strong stimulus condition for enhancing sympathetic arousal and triggering cardiovascular responses. Importantly, excessive cardiovascular response (i.e., high HR and BP increase) are related to a heightened risk of developing cardiovascular diseases, and high competitiveness trait might be a mechanism enhancing this relation (Glass et al., 1980; Sherwood et al., 1989; Shahidi et al., 1991; Harrison et al., 2001; Ricarte et al., 2001; Matsumura et al., 2011). Indeed, competitiveness is a core feature of the “Type A behavior” pattern (also called “Type A coronary-prone behavior”), a set of behavioral dispositions characterized by time urgency, impatience, restlessness, hostility, hyperalertness, and job involvement (Friedman and Rosenman, 1974) that has been associated with increased risk for coronary heart disease (Haynes and Feinleib, 1982; Orth-Gomer and Unden, 1990). Under competitive conditions, individuals classified as Type A have been reported to display larger cardiovascular responses (i.e., elevated HR and BP reactions) to laboratory and environmental challenges (Van Egeren et al., 1978; Dembroski et al., 1979; Glass et al., 1980; Chida and Hamer, 2008). In the majority of the studies focusing on the relation between competitiveness and increased cardiovascular risk, competition has been manipulated using psychosocial tasks, the most common being playing a game while competing against another person to win a prize (e.g., money). During such tasks, the participants are induced to compete against each other (or with a stooge opponent), while cardiovascular responses are monitored (Adam et al., 2015).

Intriguingly, competition during biofeedback (BF) can be used to highlight the effects of competition on the same physiological response that is going to be changed (Stegagno and Vaitl, 1979). BF is a well-known autoregulation procedure that allows the individual to control, through feedback, his/her own physiological functions, including the cardiovascular functions (Obrist et al., 1975; Williamson and Blanchard, 1979; Schwartz and Andrasik, 2017). BF can be used as a procedure to exploit competition as a motivational factor for challenging individuals to change their physiological activity (Stegagno and Vaitl, 1979; Stern and Elder, 1982; Shahidi and Salmon, 1992; Palomba and Stegagno, 1993). In an early study, a competitive BF procedure directly aimed at controlling HR was developed: participants were required to increase their HR to a greater extent than the challenger (in fact an experimental manipulation). Each participant received information on his/her HR, plus visual feedback (a red light) indicating when and for how long his/her own HR was higher compared to the challenger’s HR. The competitive situation resulted in a higher HR increase compared to the non-competitive one. Also, the respiration rate, muscle tension, and systolic blood pressure (SBP) increased significantly during the competition, reflecting general physiological arousal. It could be argued that the positive results were sustained by the synergism between the task requests (i.e., increase HR) and the motivational disposition induced by the competitive condition (Stegagno and Vaitl, 1979).

Competitive BF has also been applied to obtain a decrease in HR. Specifically, participants were rewarded for producing a physiological directional change (i.e., HR reduction) incompatible with the general psychophysiological activation induced by competition. Results showed that participants could reduce their HR under competition, although this reduction was smaller compared to the non-competitive one. Remarkably, respiration rate, muscle tension, and SBP showed no modifications during the competitive HR-BF, suggesting the idea that BF could counteract the psychophysiological activation usually elicited by competition (Palomba and Stegagno, 1993).

Competing to achieve deep relaxation was also tested. Participants received an HR-BF and were told that they would earn a monetary reward based on their ability to relax. Individuals with high competitive traits were able to reduce their HR even more in the competitive condition compared to not competitive ones. When striving to excel, individuals high in competitiveness could be more motivated to produce the expected performance, even when the performance implies a reduction in physiological activation, a response presumably incompatible with the effects of competition (Shahidi and Salmon, 1992). This is intriguing given that competitive situations are usually associated with a greater psychophysiological activation, which, in turn, has been consistently implicated in the risk to develop cardiovascular diseases, such as hypertension, coronary heart disease, heart failure, and myocardial infarction (Treiber et al., 2003; Kupper et al., 2015).

In health settings, BF is generally employed as an intervention to *decrease* psychophysiological activation, for example, through reducing HR (Hauri, 1975; Sharpley, 1989; Huang and Luk, 2015; Brown and Bray, 2019). In the early 80s, BF to improve respiratory sinus arrhythmia (RSA-BF), also called HRV-BF, was developed to target specifically the parasympathetic nervous system (Lehrer, 2013). During RSA-BF, individuals learn to synchronize the respiratory rate with variations in HR, in order to maximize RSA and the cardiac vagal control (Lehrer et al., 2000; Schwartz and Andrasik, 2017). The beneficial effects of RSA-BF have been hypothesized to be underlain different mechanisms. First, RSA-BF is linked to an increase in parasympathetic autonomic modulation (Lehrer and Gevirtz, 2014). It has been proposed that the mechanical effects of slow breathing stimulate the vagal nerve both phasically and tonically (Gerritsen and Band, 2018). Also, the synchronized oscillation in respiratory rate and HR stimulates the baroreflex (Vaschillo et al., 2006, 2011). Furthermore, positive effects have been sown on the respiratory system, and specifically an increase in gas exchange efficiency (Grossman and Taylor, 2007). More recently, some indirect anti-inflammatory effects of RSA-BF have been suggested (Gevirtz, 2013; Noble and Hochman, 2019; Lehrer et al., 2020).

RSA-BF is of particular relevance given that reduced cardiac vagal control (measured as low HRV or RSA) has been linked to several medical (Patron et al., 2012; Zhou et al., 2016; Benichou et al., 2018; Carvalho et al., 2018) and psychopathological conditions (Clamor et al., 2016; Cheng et al., 2019; Koch et al., 2019). Indeed, RSA-BF has been shown to effectively improve cardiac vagal control and, in turn, lower anxious and depressive symptoms (Karavidas et al., 2007; Gevirtz, 2013; Patron et al., 2013; Goessl et al., 2017; Caldwell and Steffen, 2018) and improve athletic performance, sleep, and quality of life (Zaccaro et al., 2018; Lehrer et al., 2020). Furthermore, RSA-BF was found to be effective in increasing cardiac vagal control and reducing SBP in a group of high-status-position managers (Munafò et al., 2016).

In addition to the mechanisms previously cited, the positive effects of RSA-BF on psychophysiological flexibility and emotions could involve improved functional connectivity between cortical brain areas. According to the neurovisceral integration model (Thayer and Lane, 2000, 2009), the heart is bidirectionally linked to areas in the prefrontal cortex through the vagus nerve and subcortical areas included in the central autonomic network (Benarroch, 1993, 1997). Mather and Thayer (2018) proposed that increasing RSA through RSA-BF could promote functional connectivity between brain regions involved in emotion regulation, such as the medial prefrontal cortex and the amygdala. Supporting this hypothesis, higher HRV has been reported to correlate with higher prefrontal cortex activity (Thayer et al., 2012; Chang et al., 2013; Patron et al., 2019), greater functional connectivity between the medial prefrontal cortex and the amygdala (Jennings et al., 2016) and with improved emotional regulation and psychological health (Hansen et al., 2003; O’Connor et al., 2007; Lane et al., 2013; Gillie et al., 2014).

Despite the positive effects of RSA-BF on cardiac vagal control, no study, to date, has applied competition to RSA-BF to examine whether competing to increase RSA could boost the motivation, leading to a greater reduction in psychophysiological activation. It could be argued that BF aiming at competing to increase RSA could improve parasympathetic modulation on the heart to a greater extent than non-competitive RSA-BF. This, in turn, could counteract the psychophysiological activation usually linked to competition. In the present study, managers in highly competitive job contexts and characterized by high competitiveness traits were randomly assigned to five sessions of competitive or non-competitive RSA-BF training. Participants in the competition group underwent BF in couples and were requested to achieve a better RSA than their competitors, while participants in the non-competition group were asked to enhance their RSA as much as they could. First, it was hypothesized that participants in the competition group would be able to enhance their RSA (i.e., increase cardiac vagal control) to a greater extent than participants in the non-competition (control) condition. Second, it was hypothesized that competing to improve RSA would counteract the psychophysiological activation usually linked to competition, leading to a reduction of HR, SBP, and skin conductance level (SCL).

## Materials and Methods

### Participants

The present study enrolled 30 managers from private (banking group, manufacturing industries, and media) and public (health service, education system, local government, and military) companies in the northeastern region of Italy. A power analysis was conducted to determine the sample size for repeated measure of analysis of variance (ANOVA) with an effect size *F* = 0.30, a correlation among repeated measures of *r* = 0.62 and a power = 0.95. Participants were recruited through advertisements in the newsletter of the association of the General Confederation of Italian Industry (Confindustria) and voluntarily participated in this study. Participants were in charge either of the whole company (manager) or departments in organization managing (middle manager), subject to a highly competitive work environment. Part of the sample from a previously published report (Munafò et al., 2016) was included in the present study. All participants were males, aged 35–67 years (mean ± SD age = 49.30 ± 8.15), with a high-level education (mean ± SD education years = 17.60 ± 2.50), and they were all actively employed, with no precedent heart problems or other chronic mental or neurological diseases. None of the participants were taking medications influencing HR (e.g., beta-blockers), tranquilizers, or antidepressants.

Participants were instructed about the study procedure and gave written informed consent. After the assessment evaluation, they were randomly assigned to the Competition (*n* = 14) or Non-Competition (Control; *n* = 16) group. The study was carried out in accordance with the Declaration of Helsinki, and the study protocol was approved by the Ethical Committee of the Psychology section of the University of Padova (prot. No. 1159).

### Measurements and Apparatus

A semi-structured interview was conducted to collect sociodemographic (age and education) and health behavior data, including weight, height, physical activity, sleep, family history of hypertension, and cardiovascular diseases as well as medication intake (including medications influencing cardiac activity and psychotropic drugs).

The Jenkins Activity Survey (JAS; Jenkins et al., 1979) was administered to assess competitiveness traits. The JAS is a self-report measure containing 54 items investigating the way of responding to situations that should elicit Type A behavior in the susceptible individual (e.g., having to wait in long lines or to work with a slow partner). The JAS has four major components: Type A scale, factor S (speed impatience), factor J (job involvement), and factor H (hard-driving and competitive).

The Social Interaction Anxiety Scale (SIAS; Mattick and Clarke, 1998) was administered to assess the fear of general social interaction. The SIAS is a self-report questionnaire that includes 20 items describing the typical cognitive, affective, or behavioral reaction to different situations requiring interaction with other persons (one or more). Each item is rated on a scale from 0 to 4, which indicates to what extent the statements reflect the respondent characteristics. Total scores range from 0 to 80, higher scores reflect higher levels of social interactional anxiety.

The State and Trait Anxiety Inventory (STAI Y1 and STAI Y2) (Spielberger et al., 1983; Pedrabissi and Santinello, 1989) was administered to assess self-reported state (Y1) and trait (Y2) anxiety symptoms. The scores range between 20 and 80; higher scores represent higher long-lasting and persistent anxiety.

The Center for Epidemiological Study of Depression scale (CES-D) (Radloff, 1977; Fava, 1982) is a 20-item self-report questionnaire designed to measure the presence of common symptoms of depression over the previous week. Each item is rated on a four-point Likert scale and scores range from 0 to 60, higher scores indicating greater depressive symptoms.

### Physiological Measures

Blood volume pulse (BVP) was recorded by a photoplethysmographic detection sensor (BVP-Flex/Pro) attached to the right ring finger. Photoplethysmography (PPG) is a more convenient and less invasive alternative to the gold standard electrocardiogram. Several studies have reported that HRV indexes calculated from PPG signal and gold standard electrocardiographic recording are highly correlated (Lu et al., 2008, 2009; Gil et al., 2010; Jeyhani et al., 2015; Pinheiro et al., 2016; Menghini et al., 2019). PPG recordings have satisfactory accuracy in healthy individuals (Pinheiro et al., 2016) during resting conditions in the absence of motion (Schäfer and Vagedes, 2013; Menghini et al., 2019).

After recording the raw BVP, the signal was visually inspected and corrected for movement artifacts, and ectopic beats were detected and eliminated. Then to obtain the interbeat intervals (IBIs) series, heartbeats were automatically identified by an algorithm based on the detection of the point of maximum deviation in the BVP signal. Then IBIs series were exported in the Kubios-HRV 2.0 (Kuopio, Finland) software where an additional artifacts correction was run applying a piecewise cubic spline interpolation method that generates missing or corrupted values into the IBIs series.

Respiration rate was recorded employing a respiration belt with strain gauges/tube filled with conduction fluid (Respiration-Flex/Pro sensor) worn around the participant’s abdomen. The software calculated the respiration rate from differences in the abdomen expansion in the raw signal waveform. The specific respiration range for each participant was calculated (i.e., maximum respiration rate *minus* minimum respiration rate, expressed in cycles/min), and converted in Hz (i.e., from cycles/min to cycles/s).

RSA was calculated through HRV analysis. Specifically, HRV is the physiological variation in the intervals between heartbeats, and most importantly, some indexes of HRV [e.g., rMSSD and the power in the high-frequency (HF) band)] have been shown to be reliable measures of the modulation of the parasympathetic branch of the autonomic nervous system on the heart in response to both internal and external challenges. In line with current recommendations (Laborde et al., 2017), the most common time- and frequency-domain HRV indexes were calculated and analyzed. Specifically, SDNN was calculated, which displays the cyclic components responsible for the total HRV. rMSSD was also computed, which is highly sensitive to the fluctuation of high-frequency HRV and is considered an index of vagal control on the heart. Moreover, rMSSD has been shown as relatively independent of respiration rate influences (Hill et al., 2009). The percentage of successive normal sinus beat to beat intervals more than 50 ms (pNN50) was computed, as it indicates cardiac vagal control (Berntson et al., 1993; Malik et al., 1996; Hill et al., 2009; Laborde et al., 2017). In the frequency domain, the power spectrum in the very low-frequency (VLF; from 0 to 0.04 Hz), in the low-frequency (LF; from 0.04 to 0.15 Hz), and in the high-frequency band (HF; 0.15–0.40 Hz) were obtained and logarithmically transformed to normalize their distribution (Malik et al., 1996).

Since BF training was specifically focused on slow breathing and RSA, which is a cardiorespiratory phenomenon characterized by inter-beat intervals fluctuations occurring in phase with respiration (Grossman and Taylor, 2007), RSA was also computed. RSA is specifically considered to display the rhythmic increase and decrease of cardiac vagal efferent effects upon the sinoatrial node that are linked to respiratory frequency (Eckberg, 2003; Yasuma and Hayano, 2004; Laborde et al., 2018). Since RSA is modulated by physiological mechanisms that comprehend the interaction between cardiac and respiratory responses (Grossman, 1983), respiration can confound the relation between cardiac vagal control and RSA (Gevirtz and Lehrer, 2003; Grossman and Taylor, 2007). Therefore, to acquire a more reliable measure of cardiac vagal control, RSA was obtained, controlling for the respiration rate of each participant. RSA was calculated as the power spectrum of the IBIs series occurring within the specific respiration rate range for each participant. Specifically, a Fast Fourier Transformation was applied to the variation of IBIs occurring within the specific respiration rate range for each participant (Aysin and Aysin, 2006; Grossman and Taylor, 2007). RSA values were expressed in ms^2^.

SBP and diastolic blood pressure (DBP) were recorded on the left arm. Three readings were taken at rest after adaptation to the laboratory at intervals of 1 min, and averaged, according to the recommendations for BP measurement of the American Heart Association (Pickering et al., 2005).

SCL was recorded employing two Ag/AgCl surface electrodes applied on the first and middle fingers of the right hand (Skin conductance-Flex/Pro sensor) (Fowles et al., 1981). The probe signal was constant voltage (0.5 V), and no conductive paste was applied on the skin. SCL, which is a measure of tonic electrodermal activity, has been widely used as an index of sympathetic nervous system activation that also reflects the level of psychophysiological activation (Boucsein, 2012; Boucsein et al., 2012). Specifically, the SCL signal recorded was visually examined for the occurrence of artifacts and non-specific skin conductance responses and manually corrected. Then, SCL was computed as the mean of SCL measurements across the non-artifactual recording.

BVP, respiration, and SCL were continuously recorded using a FlexComp Infiniti^TM^ encoder, which is a computerized recording system approved by the US Food and Drug Administration and visualized through the BioGraph Infiniti software (Thought Technology Ltd., Montreal, QC, Canada). Data were processed via a 14-bit analog-to-digital converter with a sampling rate of 256 Hz (bandwidth DC – 64Hz) and stored for analysis in a personal computer (DELL VOSTRO notebook, Intel Core^TM^ 2). SBP and DBP were recorded by a validated automatic wrist device (NAIS EW272, Matsushita Electrics Works Italia S.r.l.).

### Assessment

All managers underwent the same assessment protocol before the first RSA-BF session (i.e., pre-training) and approximately 2 weeks after the end of the fifth RSA-BF session (i.e., post-training), in a laboratory purposely set up at participant’s worksite. Before each session, participants were asked to abstain from alcohol, caffeinated beverages, and smoking for the 3 h preceding psychophysiological recordings. Self-report questionnaires (SIAS, STAI Y1, STAI Y2, and CES-D) were administered individually by a trained psychologist blind to the participant’s group assignment (Competition or Control group). Then, participants were invited to sit on a comfortable armchair, in a quiet, dimly lit room at a constant temperature (about 21°C). No support for the legs was employed to avoid the possible confounding effect of body position on cardiac activity. Before starting the physiological assessment, all the participants were informed of the sensors attached and the respective physiological measures being monitored (i.e., BVP, respiration rate, SCL, and BP). BVP signal was then analyzed to calculate HRV and RSA indexes. After the sensors’ placement and adaptation to the laboratory (10 min), SBP and DBP were measured. Then the recording of BVP, respiration, and SCL was carried on over 4 min at rest, and SBP and DBP were measured again at the end of the physiological recording. To note, all the physiological measures analyzed and included in the study were recorded in resting conditions during the pre-training assessment (before the first RSA-BF session) and the post-training assessment (about 2 weeks after the fifth RSA-BF session). Participants were asked to breathe normally. After the pre-training assessment, participants were randomly assigned to either the Competition or the Control group.

### Training

The training consisted of five weekly sessions, each lasting about 40 min, performed in the same laboratory of assessment. All participants were asked to abstain from alcohol, caffeinated beverages, and smoking for the 3 h preceding each BF session. RSA-BF was aimed at increasing RSA and, therefore, at opposing autonomic dysregulation, especially vagal inhibition associated with stress (Lehrer et al., 2000). Before starting the first RSA-BF session, all the managers were informed about the feedback system, and they were told that augmenting the amplitude of HR changes in phase with breathing would increase RSA. Then, instructions similar to those proposed in Lehrer’s et al. protocol (2000) were given to all participants. Specifically, they were told to try to breathe in phase with their HR, such that when the HR accelerate, they had to start inhaling, and when the HR decelerate, they had to exhale. Also, they were instructed to breathe so that their abdomen expanded during inhalation and contracted during exhalation and, more importantly, to breathe out slower than they breathed in. Finally, they were asked to breathe in through their nose and breathe out through pursed lips. After the sensors’ placement, the BF session started with a resting period of 3 min followed by two 6 min BF trials, spaced out by 1 min at rest. The BF session ended with 3 min at rest. Feedback was provided to all participants using the same instruments used for psychophysiological assessment, on a 15-inch PC display positioned in front of them at a distance of 50 cm. RSA feedback consisted of an HR beat-to-beat tachogram (i.e., beats/min) superimposed over the abdominal respiration signal on the same axis. Participants were required to synchronize HR and abdominal respiration until the two signals covaried in phase, thus leading to the maximal amplitude of RSA. The online moving feedback display (the graph representing the tachogram and abdominal respiration curves) was updated at successive 30 s periods. During each RSA-BF session, participants were reminded not to breathe too deeply to avoid hyperventilation. No pacing stimulus was provided during the training sessions.

**FIGURE 1:**
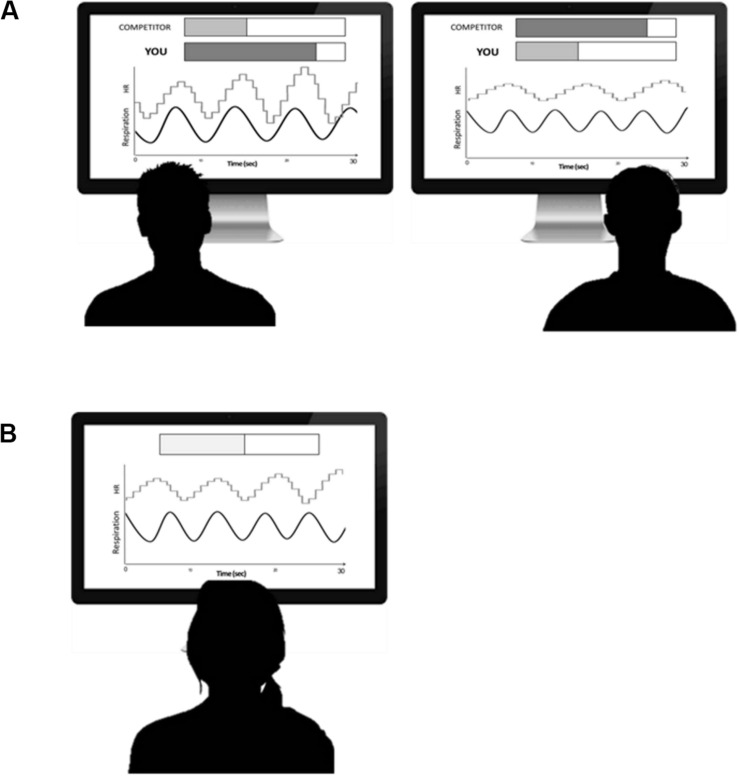
**(A)** Schematic representation of one session of competition biofeedback. On the screens (duration 30 s) are representations of abdominal breathing (black line) and heart rate tachogram (gray line). Also, two bars were included on the screen to represent feedback for the subject’s (“YOU”) and competitor’s (“COMPETITOR”) performance. The participant was asked to synchronize heart rate with abdominal breathing better than his/her competitor. **(B)** Schematic representation of one session of Control Biofeedback. On the screen (duration 30 s) are representations of abdominal breathing (black line) and heart rate tachogram (gray line). Also, one bar was included on the screen to represent feedback for the subject’s performance. The participant was asked to synchronize heart rate with abdominal breathing as much as he/she could.

Participants in the Competition group underwent the RSA-BF training in couples [paired for age, body mass index (BMI), and physical activity level) and were requested to have a *better* performance compared to the paired challenger (i.e., increase RSA *more than* the competitor). Participants in the Competition group were presented two stepped bars increasing from left to right: one bar represented their own performance and the other reflected the competitor’s one (see Figure 1). Each step increase on the bar corresponded to 10 s RSA above the mean level of RSA as recorded during the baseline (i.e., during the first 3 min at rest). Participants competing to increase RSA were asked to increase the number of steps displayed on the bar more than the competitor.

Participants in the Control group were also trained in couples, but they had no competitive feedback: one stepped bar increasing from left to right was displayed on the screen to represent feedback for participant’s performance. Participants in the Control group were asked to increase the stepped bar as much as they could.

#### Data Reduction and Analysis

Data reduction and analyses were performed on questionnaire scores (SIAS, STAI Y1, STAI Y2, and CES-D) and physiological signals (i.e., RSA, HR, SDNN, rMSSD, pNN50, VLF, LF, HF, SBP, and DBP, respiration rate, and SCL) recorded over a 4 min period at rest during pre- (i.e., before the first RSA-BF session) and post-training (about 2 weeks after the end of the fifth RSA-BF session) sessions.

Whether a variable resulted not normally distributed from the Shapiro-Wilk test, a log transformation was applied for data normalization. For this reason, pre- and post-training RSA and SCL were log-transformed. The mean respiration rate was calculated over 4 min at rest during pre- and post-training assessment sessions. SBP and DPB were separately averaged across the three recordings during pre- and post-training sessions.

Student’s *t*-tests for independent groups were performed to compare age, education, BMI, sleep time, and scores on JAS scales in the two groups (Competition and Control). χ^2^s were calculated to test differences between groups in sleep disorders, smoking, physical activity, family history of hypertension, and cardiovascular disease.

A series of repeated measure ANOVA, with Group (Competition and Control) as a between-subjects factor, and Time (pre- and post-training), as a within-subjects factor were performed on questionnaires scores (SIAS, STAI Y1, STAI Y2, and CES-D) and all physiological measures (RSA, HR, SDNN, rMSSD, pNN50, VLF, LF, HF, SBP, DBP, respiration rate, and SCL). Moreover, to evaluate whether the modification in RSA after RSA-BF training was clinically relevant, percent improvement index was calculated with the following formula [(log(RSA)post-training – log(RSA)pre-training)/log(RSA) pre-training ^∗^100] (Blanchard and Andrasik, 1987). Then a Mann-Whitney *U*-test was run to compare the RSA percent improvement index in the two groups (Competition and Control). Partial eta-squared (η*_*p*_*^2^) was reported as a measure of the effect size. Significant interactions (*p* < 0.05) were followed by Tukey *post hoc* comparisons to identify specific differences. All analyses were performed using Jamovi version 0.9 (Şahin and Aybek, 2019). A *p* < 0.05 was considered statistically significant.

## Results

### Sociodemographic and Health Behavior Data

Student’s *t*-tests for independent groups and chi-square analyses revealed no group differences for age, education, BMI, family history of hypertension, cardiovascular disease, physical activity, reported sleep time, sleep disorders, and competitiveness traits (JAS scores; all *p’s* > 0.296; see Table 1).

**TABLE 1:** Sociodemographic characteristics, health behaviors, and JAS scores of participants assigned to Competition and Control groups.

Participants’ characteristics	Competition (*n* = 14)	Control (*n* = 16)	*p*
Age (year)	48.86 (7.12)	49.69 (9.17)	0.786
Education (years)	17.50 (2.59)	17.69 (2.50)	0.842
BMI (Kg/m^2^)	26.26 (2.88)	26.73 (3.72)	0.706
Physical activity (none/occasional/regular)	3 (21)/7 (50)/4 (29)	5 (31)/4 (25)/7 (44)	0.366
Sleep time (hours)	6.82 (0.54)	6.78 (0.98)	0.893
Sleep disorders (N, %)	7 (50)	11 (69)	0.296
Family history of hypertension (N, %)	6 (43)	7 (44)	0.961
Family history of cardiovascular disease (N, %)	7 (50)	9 (56)	0.732
JAS – Speed Impatience	216.86 (80.12)	243.63 (60.54)	0.307
JAS – Job Involvement	260.79 (29.97)	252.06 (29.24)	0.427
JAS – Hard-driving and Competitive	128.21 (32.68)	127.44 (35.83)	0.951

*Data are M (SD) of continuous and N of categorical variables. BMI, Body Mass Index; JAS, Jenkins Activity Survey.*

### Questionnaires Scores

Repeated measures ANOVAs on questionnaires scores revealed a significant reduction in fear of social interaction, state and trait anxiety, and depressive symptoms from pre- to post-training in both groups, as shown by the significant Time main effects [SIAS: *F*_(1, 28)_ = 9.13; *p* = 0.005; η^2^*_*p*_* = 0.25; STAI Y1: *F*_(1, 28)_ = 4.73; *p* = 0.038; η^2^*_*p*_* = 0.14; STAI Y2: *F*_(1, 28)_ = 12.41; *p* = 0.001; η^2^*_*p*_* = 0.31; CES-D: *F*_(1, 28)_ = 5.82; *p* = 0.023; η^2^*_*p*_* = 0.17; see Tables 2, 3]. No significant main Group effect nor Group × Time interaction emerged for these measures (all *p*’s > 0.136).

**TABLE 2:** Psychophysiological and psychological indexes from pre- to post-training in participants who underwent competition RSA-BF and controls.

Variable	Pre-training	Post-training
**SIAS**		
Competition	19.07 (8.69)	16.00 (7.75)
Control	14.06 (8.39)	12.38 (7.11)
**STAI Y1**		
Competition	36.07 (6.57)	33.07 (7.21)
Control	33.00 (4.89)	30.25 (8.38)
**STAI Y2**		
Competition	34.93 (5.64)	32.64 (4.63)
Control	37.56 (8.02)	34.50 (7.56)
**CES-D**		
Competition	9.14 (3.74)	7.14 (2.32)
Control	11.19 (5.14)	8.69 (5.59)
**RSA (log[ms^2^])**		
Competition	1.90 (0.63)	2.87 (0.75)
Control	2.15 (0.70)	2.51 (0.58)
**HR (bpm)**		
Competition	71.09 (10.20)	65.84 (8.61)
Control	73.22 (11.74)	67.07 (8.26)
**SDNN (ms)**		
Competition	30.06 (17.21)	55.98 (25.73)
Control	28.55 (20.46)	39.18 (25.46)
**rMSSD (ms)**		
Competition	25.29 (16.46)	49.31 (23.12)
Control	24.38 (20.25)	31.94 (21.59)
**pNN50**		
Competition	6.89 (9.23)	13.07 (12.00)
Control	5.45 (12.62)	7.15 (12.69)
**VLF (log[ms^2^])**		
Competition	3.48 (0.93)	4.31 (0.97)
Control	3.16 (1.25)	3.54 (1.77)
**LF (log[ms^2^])**		
Competition	5.95 (0.95)	7.20 (1.30)
Control	5.63 (1.40)	6.22 (1.60)
**HF (log[ms^2^])**		
Competition	4.92 (1.20)	5.97 (1.29)
Control	4.99 (1.29)	5.28 (1.24)
**SBP (mmHg)**		
Competition	125.18 (11.21)	123.14 (14.93)
Control	130.66 (14.06)	121.44 (9.07)
**DBP (mmHg)**		
Competition	79.32 (8.37)	79.54 (9.02)
Control	82.75 (9.11)	80.59 (7.83)
**Respiration rate (breath/min)**		
Competition	15.60 (2.99)	11.28 (4.15)
Control	13.93 (2.64)	12.01 (3.64)
**SCL (log[μMho])**		
Competition	0.41 (0.24)	0.36 (0.18)
Control	0.43 (0.17)	0.27 (0.13)

*Data are M (SD). SIAS, Social Interaction Anxiety Scale; STAI Y1, State and Trait Anxiety Inventory form Y1; STAI Y2, State and Trait Anxiety Inventory form Y2; CES-D, Center for Epidemiological Study of Depression scale; RSA, respiratory sinus arrhythmia; HR, heart rate; SDNN, standard deviation of normal sinus beat to beat intervals; rMSSD, root mean square of the successive differences between adjacent heartbeats; pNN50, percentage of successive normal sinus beat to beat intervals more than 50 ms; VLF, power in the very low frequency; LF, power in the low frequency; HF, power in the high frequency; SBP, systolic blood pressure; DBP, diastolic blood pressure; SCL, skin conductance level.*

**TABLE 3:** Results of ANOVAs on questionnaires scores and physiological data from pre- to post-training in participants in the competition and control groups.

	Time main effects			Group main effects			Time × Group interactions		
Variable	*F*_(1, 27)_	*P*	η^2^*_*p*_*	*F*_(1, 27)_	*p*	η^2^*_*p*_*	*F*_(1, 27)_	*p*	η^2^*_*p*_*
SIAS	9.13	0.005	0.25	2.35	0.136	0.08	0.77	0.387	0.03
STAY1	4.73	0.038	0.14	1.89	0.180	0.06	0.01	0.925	0.001
STAI Y2	12.41	0.001	0.31	0.93	0.344	0.03	0.26	0.613	0.01
CES-D	5.82	0.023	0.17	1.79	0.192	0.06	0.07	0.791	0.001
RSA (log[ms^2^])	42.47	<0.001	0.60	0.05	0.822	0.001	8.78	0.006	0.24
HR (bpm)	10.91	0.003	0.28	0.28	0.599	0.01	0.07	0.796	0.002
SDNN (ms)	37.82	<0.001	0.19	1.42	0.244	0.05	6.62	0.016	0.19
rMSSD (ms)	28.23	<0.001	0.50	1.76	0.196	0.06	7.67	0.010	0.22
pNN50	5.75	0.023	0.17	0.85	0.365	0.03	1.86	0.184	0.06
VLF (log[ms^2^])	3.92	0.058	0.12	2.28	0.142	0.08	0.51	0.481	0.02
LF (log[ms^2^])	22.31	<0.001	0.44	2.07	0.161	0.07	2.85	0.103	0.09
HF (log[ms^2^])	9.50	0.005	0.25	0.60	0.444	0.02	3.05	0.092	0.10
SBP (mmHg)	7.08	0.013	0.20	0.22	0.645	0.01	2.88	0.101	0.09
DBP (mmHg)	0.27	0.605	0.01	0.78	0.384	0.03	0.41	0.528	0.01
Respiration rate(breath/min)	24.38	<0.001	0.47	0.19	0.664	0.01	3.62	0.067	0.11
SCL (log[μMho])	13.24	0.001	0.32	0.39	0.540	0.01	3.16	0.086	0.10

*SIAS, Social Interaction Anxiety Scale; STAI Y2, State and Trait Anxiety Inventory form Y2; CES-D, Center for Epidemiological Study of Depression scale; RSA, respiratory sinus arrhythmia; HR, heart rate; SDNN, standard deviation of normal sinus beat to beat intervals; rMSSD, root mean square of the successive differences between adjacent heartbeats; pNN50, percentage of successive normal sinus beat to beat intervals more than 50 ms; VLF, power in the very low frequency; LF, power in the low frequency; HF, power in the high frequency; SBP, systolic blood pressure; DBP, diastolic blood pressure; SCL, skin conductance level.*

### Physiological Data

**FIGURE 2:**
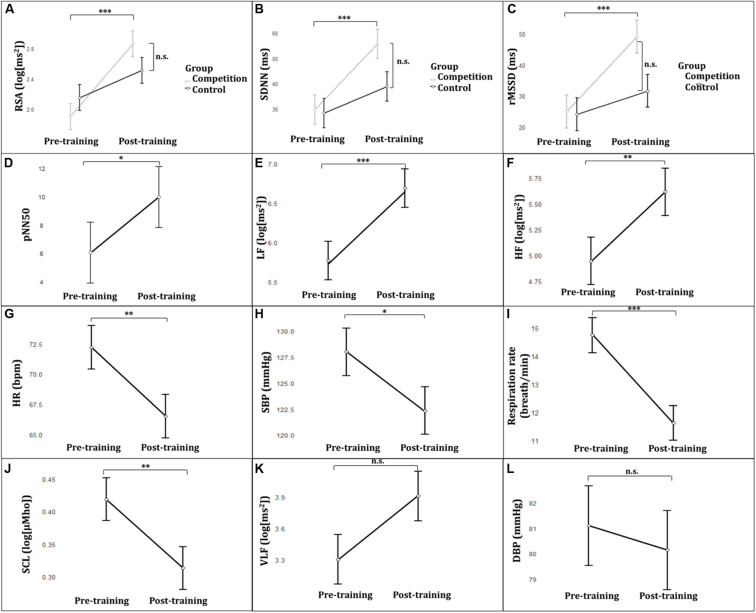
**(A)** RSA in the Competition and Control group frompre-to post-training. **(B)** SDNN in the Competition and Control group from pre-to post-training. **(C)** rMSSD in the Competition and Control group from pre-to post-training. **(D)** pNN50 from pre- to post-training. **(E)** LF from pre- to post-training. **(F)** HF from pre- to post-training. **(G)** HR from pre- to post-training. **(H)** SBP from pre- to post-training. **(I)** Respiration rate from pre- to post-training. **(J)** SCL from pre- to post-training. **(K)** VLF from preto post-training. **(L)** DBP from pre- to post-training. Error bars represent the standard error of the mean. ^∗^*p* < 0.05; ^∗∗^*p* < 0.01; ^∗∗∗^*p* < 0.001. RSA, respiratory sinus arrhythmia; SDNN, standard deviation of normal sinus beat to beat intervals; rMSSD, root mean square successive difference of normal sinus beat to beat intervals; pNN50, percentage of successive normal sinus beat to beat intervals more than 50 ms; LF, power in the low frequency; HF, power in the high frequency; HR, heart rate; SBP, systolic blood pressure; SCL, skin conductance level; VLF, power in the very low frequency; DBP, diastolic blood pressure.

The ANOVA on RSA at rest showed a Group × Time interaction [*F*_(1, 27)_ = 8.78; *p* = 0.006; η^2^*_*p*_* = 0.24; see Tables 2, 3 and Figure 2A). *Post hoc* comparisons yielded a significant RSA increase from pre- to post-training in the Competition group (*p* < 0.001), whereas in the Control group pre- to post-training comparison did not reach significance (*p* = 0.066). Post-training comparison between the Competition and Control group was not significant (*p* = 0.479). A significant main effect of Time emerged [*F*_(1, 27)_ = 42.47; *p* < 0.001.; η^2^*_*p*_* = 0.60], revealing higher resting RSA during post-training assessment compared to pre-training. No significant main effect of Group emerged (*p* = 0.822)^1^.

The Mann-Whitney U test on percent improvement index revealed that managers in the Competition group after RSA-BF had a greater percent improvement index (57%), than the Control group (27%) (Mann-Whitney *U* = 51.00; *p* = 0.010).

The ANOVA on SDNN revealed a significant Group × Time interaction [*F*_(1, 27)_ = 6.62; *p* = 0.016; η^2^*_*p*_* = 0.19; see Tables 2, 3 and Figure 2B). Tukey *post hoc* comparisons displayed a significant SDNN increase from pre- to post-training in the Competition group (*p* < 0.001), whereas the comparison between pre- and post-training in the Control group did not reach statistical significance (*p* = 0.064). Post-training comparison between the Competition and Control group was not significant (*p* = 0.194). Main Time effect yielded a significant increase in SDNN from pre- to post-training [*F*_(1, 28)_ = 37.82; *p* = ≤ 0.001; η^2^*_*p*_* = 0.19]. No significant main effect of Group emerged (*p* = 0.244).

The ANOVA on rMSSD showed a significant Group × Time interaction [F_(1, 27)_ = 7.67; *p* = 0.010; η^2^*_*p*_* = 0.22; see Tables 2, 3 and Figure 2C]. *Post hoc* comparisons yielded a significant rMSSD increase from pre- to post-training in the Competition group (*p* < 0.001), whereas the comparison between pre- and post-training in the Control group was not significant (*p* = 0.267). Post-training comparison between the Competition and Control group was not significant (*p* = 0.113). Main Time effect yielded a significant increase in rMSSD from pre- to post-training [*F*_(1, 28)_ = 28.23; *p* = ≤ 0.001; η^2^*_*p*_* = 0.50]. No significant main effect of Group emerged (*p* = 0.196).

Also, a significant Time main effect emerged showing an increase in pNN50, LF and HF [pNN50: *F*_(1, 28)_ = 5.75; *p* = 0.023; η^2^*_*p*_* = 0.17; see Figure 2D; LF: *F*_(1, 28)_ = 22.31; *p* = 0.001; η^2^*_*p*_* = 0.44; see Figure 2E; HF: *F*_(1, 28)_ = 9.50; *p* = 0.005; η^2^*_*p*_* = 0.25; see Tables 2, 3 and Figure 2F).

A significant reduction in HR, SBP, respiration rate, and SCL occurred from pre- to post-training for both groups, as shown by the significant Time main effects [HR: *F*_(1, 28)_ = 10.91; *p* = 0.003; η^2^*_*p*_* = 0.28; see Figure 2G; SBP: *F*_(1, 28)_ = 7.08; *p* = 0.013; η^2^*_*p*_* = 0.20; see Figure 2H; respiration rate: *F*_(1, 28)_ = 24.38; *p* < 0.001; η^2^*_*p*_* = 0.47;, see Figure 2I; SCL: *F*_(1, 28)_ = 13.24; *p* = 0.001; η^2^*_*p*_* = 0.32;, see Tables 2, 3 and Figure 2J]. No other significant effects emerged (all *p*’s > 0.058; see Figures 2K,L).

## Discussion

The present study examined whether managers characterized by high competitiveness traits who were asked to compete to enhance their own cardiac vagal control through BF would achieve a greater improvement in RSA in comparison to managers undergoing a traditional (non-competitive) RSA-BF. Moreover, competing to improve RSA was expected to counteract the psychophysiological activation commonly linked to competition, leading to a reduction in HR, SBP, and SCL.

One major result of the present study was that managers in the Competition group significantly increased RSA from pre- to post-training. Importantly, managers in the Competition group specifically showed a consistent increase in indexes reflecting total HRV (i.e., SDNN) and greater cardiac vagal control (i.e., RSA and rMSSD). Additionally, to evaluate whether the modification in RSA after RSA-BF training was clinically relevant, percent improvement index was calculated. According to Blanchard and Andrasik (1987), a percent improvement index is clinically relevant when higher than 50%. In the present study, RSA percent improvement was clinically relevant only in the Competition group (57%), whereas the Control group showed a significantly lower RSA percent improvement (27%). Taken together these results support the idea that competitive BF was effective in improving cardiac vagal control to a greater extent than traditional non-competitive RSA-BF. This supports the idea that competition could have increased participants’ motivation for success. Specifically, the feedback may become more relevant for the participants when they can use it in a competitive situation to achieve better results.

Intriguingly, the literature commonly reports the link between competition and excessive sympathetic activation (i.e., increased pre-ejection period) and general psychophysiological activation (i.e., increased HR and BP) (Harrison et al., 2001; van Zanten et al., 2002). In contrast, the present results suggest a different perspective, showing that competition can be associated with an increase in parasympathetic cardiac modulation (i.e., higher RSA and rMSSD). In line with the idea that managers competing to improve cardiac vagal control were able to counteract the psychophysiological activation commonly associated with competition, managers in the competition group showed, after the training, a reduction in resting HR, SBP, and SCL comparable to that found in managers who did not compete. A previous study showed that competition was effective in improving performance during autoregulation, especially when the physiological modification requested by BF was compatible with the competition condition (i.e., accelerating HR) (Stegagno and Vaitl, 1979). In that case, a synergy between the physiological modification (i.e., to increase HR) and competition emerged together with a generalization of the effect to other cardiovascular responses possibly linked to increased psychophysiological activation (i.e., increased BP). On the contrary, when the direction of the physiological modification requested is incompatible with competition activation (e.g., compete to reduce the physiological activation), a mutual inhibition is expected between competition and autocontrol. However, results from previous studies suggest that it is possible for individuals to control an activating situation (i.e., competition) that is incompatible with the task requested by the feedback (i.e., physiological deactivation) (Shahidi and Salmon, 1992; Palomba and Stegagno, 1993).

The present results suggest that both competitive and non-competitive conditions were associated with increased HRV indexes (i.e., higher pNN50, LF, and HF from pre- to post-training) and lower psychophysiological activation (i.e., lower HR, SBP, and SCL from pre- to post-training). These findings are consistent with a previous study reporting the positive effects of RSA-BF in enhancing cardiac vagal control in highly competitive managers (Munafò et al., 2016). Also, the present results are in line with previous studies reporting reduced overall physiological arousal after RSA-BF training (Gevirtz and Lehrer, 2003; Wheat and Larkin, 2010). These results also suggest the possible contribution of RSA-BF to reducing the harmful effects of cardiovascular activation (Lehrer et al., 1997; Sherlin et al., 2009). This is noteworthy given that epidemiological studies in the general population have consistently shown that elevated levels of resting HR and SBP (even if not clinically relevant) are associated with increased risk for cardiovascular mortality (Kannel, 1996; Palatini et al., 2006; Reil and Böhm, 2007; Gu et al., 2008; Palatini, 2009).

Managers in a highly competitive job context and characterized by high competitiveness due to the elevated levels of involvement, competition, and responsibility have been shown to have a higher risk of cardiovascular disease (Kivimäki et al., 2002; Belkic et al., 2004; Backé et al., 2012). To decrease cardiovascular risk by reducing physiological arousal, occupational stress, and job strain, a wide variety of interventions including stress-management, relaxation, meditation techniques, and diaphragmatic deep breathing have been suggested (Lazarus and Folkman, 1984; Ivancevich et al., 1990; Giga et al., 2003). Whereas outcome evaluation of these interventions relied mainly on self-reporting measures (Kushnir et al., 1998) – with no objective measurement of the effectiveness in the reduction of psychophysiological activation – in the present study, physiological measures have been specifically targeted. From the present results, it could be argued that competing to increase RSA allowed participants to enhance their cardiac vagal control (i.e., RSA and rMSSD) to a greater extent than participants in the non-competition group. This, in turn, might have contributed in counteracting the psychophysiological cardiovascular activation usually found during competition, which have been proposed as one of the factors increasing cardiovascular risk.

An improvement in RSA has been linked to greater physiological flexibility and adaptive regulation to environmental challenges, as well as to psychological well-being, including anxiety and depressive symptoms reduction (Karavidas et al., 2007; Patron et al., 2013; Goessl et al., 2017; Caldwell and Steffen, 2018). The present study showed that managers competing to increase RSA reported a reduction in social anxiety, state and trait anxiety, and depressive symptoms corresponding to the reduction showed by managers undergoing traditional RSA-BF. This is in line with previous studies showing the effectiveness of RSA-BF in reducing anxiety symptoms and improving mood and psychological health (Karavidas et al., 2007; Gevirtz, 2013; Patron et al., 2013; Goessl et al., 2017; Caldwell and Steffen, 2018; Zaccaro et al., 2018; Lehrer et al., 2020). Recently it has been suggested that increasing RSA through RSA-BF could promote functional connectivity between certain brain regions involved in emotion regulation (Mather and Thayer, 2018). Future studies are warranted to verify whether the positive effects on mood and emotion regulation after RSA-BF are associated with greater brain functional connectivity.

It should be considered that in the present study participants were specifically recruited for their exposition to a competitive environment and their high competitiveness traits. The literature reports that individual high in trait competitiveness is characterized by more pronounced physiological activation to challenges (Harrison et al., 2001). Since highly competitive individuals strive to excel (e.g., Stern and Elder, 1982; Shahidi and Salmon, 1992), competitiveness could be manipulated as a motivational factor to enhance the performance, even when a reduction in physiological activation is demanded. Moreover, it has been reported that stronger motivation and better performances are observed only under appropriate competition conditions (Stanne et al., 1999). Indeed, the competition must be appropriately balanced (e.g., avoiding an excessive emphasis on winning, unequal participants matching), and participants should be able to estimate their progress relative to their opponent. In the present study, great attention was directed on setting an appropriate competition condition: the BF protocol created a fair challenging competition, providing each participant with a realistic and equal chance of winning; competitors were paired based on their age, BMI, and physical activity levels; the rules were clear and straightforward. Finally, participants could constantly assess their progress, relative to their opponent, through the feedback. It could be argued that individuals characterized by high competitiveness traits might take the most advantage from competition to motivate better self-regulation. Future studies are warranted to investigate whether competitiveness traits modulate the effectiveness of competitive BF in increasing cardiac vagal control.

The current findings should be interpreted considering some possible methodological limitations. First, this study used a relatively small sample size; therefore, the results need to be replicated to fully understand the effects of competition BF in the acquisition of autonomic regulation in individuals with high levels of competitiveness. Nonetheless, the sample was determined through a power analysis based on previous studies on RSA-BF using the same protocol (Patron et al., 2013; Munafò et al., 2016). Second, although the current study showed that RSA is modifiable through competitive BF in a short time frame, the long-term effects of competitive RSA-BF were not assessed. Future research is warranted to replicate and extend the present findings by conducting long-term follow-up studies to demonstrate first the longevity of the improvements in RSA and whether the positive effects of RSA-BF could be linked to reduced cardiovascular risk. Third, although the present study focused on the effects of RSA-BF on cardiac vagal control, and secondly on the effects of RSA-BF on psychophysiological activation as measured by HR, SBP, and SCL, no specific index of the sympathetic nervous system influence on the heart was included. Future studies including measures of cardiac sympathetic nervous system influence and specific measures of cardiac output are warranted to directly compare the possible effects of RSA-BF on both the parasympathetic and sympathetic cardiac influence.

To summarize, managers competing to improve their cardiac vagal control showed a greater increase in RSA and rMSSD than managers in the non-competitive condition. Despite competition have been consistently associated with increased psychophysiological activation, the present results yield that managers competing for improving their cardiac vagal control (by increasing RSA) were able to reduce psychophysiological activation (i.e., lower HR, SBP, and SCL) and decrease anxiety and depressive symptoms to the same extent as managers in a non-competitive condition. In conclusion, the present study suggests that individuals with high competitiveness traits may benefit from competitive conditions during BF to increase cardiac vagal control. In turn, increased cardiac vagal control may counteract the psychophysiological activation linked to competition possibly leading to better autonomic regulation and psychophysiological well-being.

## Data Availability Statement

The raw data supporting the conclusions of this article will be made available by the authors, without undue reservation, to any qualified researcher.

## Ethics Statement

The studies involving human participants were reviewed and approved by the Ethic Committee of the Department of General Psychology, University of Padua. The patients/participants provided their written informed consent to participate in this study.

## Author Contributions

DP, LS, and MM contributed conception and design of the study. MM and EP gathered the data, organized the dataset, and wrote the manuscript. SM and EP performed the statistical analysis. All authors contributed to manuscript revision, read, and approved the submitted version.

## Conflict of Interest

The authors declare that the research was conducted in the absence of any commercial or financial relationships that could be construed as a potential conflict of interest.

## Footnotes

1 A repeated measure analysis of covariance (ANCOVA) on RSA controlling for change from pre- to post-training in respiration rate as a covariate was computed. Results showed a significant Group × Time interaction [*F*_(1, 27)_ = 4.46; *p* = 0.044; η^2^*_*p*_* = 0.14]. *Post hoc* comparisons yielded a significant RSA increase from pre- to post-training in both the Competition group (*p* < 0.001) and the Control group (*p* = 0.003). Post-training comparison between the Competition and Control group was not significant (*p* = 0.958). A significant main effect of Time emerged [*F*_(1, 27)_ = 10.42; *p* = 0.003; η^2^*_*p*_* = 0.28], revealing higher resting RSA during post-training assessment compared to pre-training. No significant main effect of Group emerged (*p* = 0.776).
